# The role of APOE4 in Alzheimer’s disease: strategies for future therapeutic interventions

**DOI:** 10.1042/NS20180203

**Published:** 2019-04-18

**Authors:** Holly C. Hunsberger, Priyanka D. Pinky, Warren Smith, Vishnu Suppiramaniam, Miranda N. Reed

**Affiliations:** 1 Division of Systems Neuroscience, Research Foundation for Mental Hygiene, Inc. (RFMH)/New York State Psychiatric Institute (NYSPI), New York, NY 10032, U.S.A.; 2 Department of Psychiatry, Columbia University, New York, NY 10032, U.S.A.; 3 Department of Drug Discovery and Development, Harrison School of Pharmacy, Auburn, AL 36849, U.S.A.; 4 Center for Neuroscience Initiative, Auburn University, Auburn, AL 36849, U.S.A.

**Keywords:** Apoe, Alzheimer, Amyloid, Therapeutics, Tau

## Abstract

Alzheimer’s disease (AD) is the leading cause of dementia affecting almost 50 million people worldwide. The ε4 allele of Apolipoprotein E (APOE) is the strongest known genetic risk factor for late-onset AD cases, with homozygous *APOE4* carriers being approximately 15-times more likely to develop the disease. With 25% of the population being *APOE4* carriers, understanding the role of this allele in AD pathogenesis and pathophysiology is crucial. Though the exact mechanism by which ε4 allele increases the risk for AD is unknown, the processes mediated by APOE, including cholesterol transport, synapse formation, modulation of neurite outgrowth, synaptic plasticity, destabilization of microtubules, and β-amyloid clearance, suggest potential therapeutic targets. This review will summarize the impact of APOE on neurons and neuronal signaling, the interactions between APOE and AD pathology, and the association with memory decline. We will then describe current treatments targeting APOE4, complications associated with the current therapies, and suggestions for future areas of research and treatment.

## Introduction

As the sixth leading cause of death, killing more than breast and prostate cancers combined, Alzheimer’s disease (AD) cannot be prevented, slowed, or cured [1]. AD is a debilitating neurodegenerative disorder characterized by cognitive decline, the risk for which increases significantly with age [2]. Biologically, AD is characterized by plaques consisting of β-amyloid protein, tangles consisting of the τ protein, and neuronal loss [2]. Because AD is a multifaceted disease with both genetic and environmental risk factors, there has been little success in regard to disease-modifying treatment. This review will focus on the genetic component of sporadic AD, which represents more than 90% of AD patients.

To date, the only gene consistently found to be associated with sporadic AD is the apolipoprotein E (*APOE*) gene [3]. Discovered 44 years ago in human plasma, this gene was originally termed as the ‘arginine-rich’ protein [4]. However, it took two decades after the initial discovery to determine that APOE was also present in the brain [5]. Interestingly, humans are the only species known to produce multiple APOE isoforms [6], three of which are APOE2, APOE3, and APOE4 [7]. There is only one APOE variant in all other animal species, which most closely resembles the human e3 allele. APOE3, which arose from APOE4 during early human evolution, represents the shift from a plant-based diet to a meat-based diet where ‘meat-adaptive’ genes were and still are necessary to control higher cholesterol levels [8]. This partly explains why APOE3 carriers are less susceptible to diseases such as cardiovascular and AD, while APOE4 represents the strongest genetic risk factor for sporadic AD. Only a single amino acid difference exists between APOE3 (Cys^112^) and APOE4 (Arg^112^) resulting in a conformational change which affects binding to APOE receptors, lipids, and amyloid [9]. One copy of the *ε4* allele accelerates AD onset by 2–5 years, while two copies accelerate onset by 5–10 years [3]. The *ε2* allele is generally regarded as neuroprotective [10], however two copies of the *ε2* allele can increase the risk for cardiovascular disease [11].

In healthy physiological conditions, the APOE protein circulates through the bloodstream as part of a large lipoprotein molecule where it plays an essential role in regulating cholesterol and lipid metabolism, as well as cellular signaling and repair [12]. In the brain, astrocytes are the major source of APOE, while the liver produces APOE in the periphery [13,14]. Peripheral and brain cholesterol are independent of each other and separated by the blood–brain barrier (BBB) [15]. Of the three isoforms, APOE4 is responsible for elevating cholesterol in the brain to a greater extent than APOE3, and APOE2 carriers tend to have lower cholesterol levels due to reduced binding affinity for low-density lipoprotein (LDL) [7]. Thus, cholesterol transport may play an important role in the progression of AD. Other processes mediated by APOE include synapse formation, modulation of neurite outgrowth, synaptic plasticity, destabilization of microtubules, and β-amyloid clearance. For the past three decades, numerous clinical trials targeting amyloid pathology have failed, which suggests that additional research and treatment strategies should be explored. The following review will summarize the impact of APOE4 on neurons and neuronal signaling, the interactions between APOE and AD pathology, and association with memory decline. We will then describe current treatments targeting APOE4, why therapies are failing, and how we can improve in this area of research.

## APOE and neuronal signaling

APOE not only functions to traffic lipids through the PNS and CNS, but also interacts heavily with the tripartite synapse (presynapse, postsynapse, and astrocytes) and neuronal signaling. The best characterized neuronal signaling pathway mediated by APOE is the reelin pathway, which acts through APOE receptor 2 (APOER2). Reelin binding is important for neuronal migration, dendritic spine development, and synaptic plasticity [16]. Reelin binds to APOER2 post-synaptically and activates cystolic receptor disabled-1 (Dab1), subsequently leading to phosphorylation of NMDA receptors, calcium influx, and long-term potentiation (LTP) [17]. Unlike APOE2 and APOE3, APOE4 is detrimental to this process as it promotes cellular uptake of APOER2. This limits reelin binding, thereby causing a decrease in synaptic plasticity [18]. In addition, reelin levels are reduced with age and in AD, specifically in the entorhinal cortex (EC), which is also one of the first areas affected by AD pathology [19]. Although these studies suggest that APOE4 leads to a decrease in neuronal signaling, there are controversial findings showing that transgenic mouse models of AD and patients at risk for AD manifest early neuronal hyperactivity in the hippocampus [20–22]. Mice containing the APOE4 human gene in place of endogenous mouse *APOE* gene exhibit an increase in brain metabolism and activity in the hippocampus, most notably in the EC [23]. This neuronal hyperactivity is driven by a decrease in inhibitory tone in EC. More specifically, excitatory neurons are less responsive to GABAergic inhibitory inputs [23]. The resulting increase in brain activity may promote the spread of AD-related pathology, as previous work suggests increases in activity promotes secretion of both amyloid and τ [24–28]. Taken together, APOE4 may drive hyperactivity and increase pathology, eventually leading to neurodegeneration and hypoactivity observed at later stages of the disease.

Although most work investigating neuronal signaling interactions with APOE4 involves the post-synaptic area, recent work suggests that presynaptic release of vesicles and glutamate is also impacted by APOE4 [29]. Specifically, APOE4 neurons have difficulty converting glutamine into glutamate, which leads to a compensatory increase in vesicular glutamate transporter 1 [30]. Whether or not this has a direct impact on glutamate production and the subsequent release *in vivo* is still unknown. However, we do know that reelin signaling through presynaptic APOER2 leads to an influx of intracellular Ca^2+^, thereby increasing the fusion of vesicles to the membrane [29]. Little is known about the impact of different APOE isoforms on the presynapse, which represents an untapped area for future therapeutic targets and research.

Astrocytes are the main source of APOE in the brain and signaling through astrocytes results in synaptic pruning [31], synaptic transmission [32], and glial transmission [33]. Although research into these mechanisms is limited, studies show that the APOE4 isoform limits pruning likely through a variety of phagocytic receptors (i.e., MEGF10, MERTK, AXL, INTEGRIN, α5β5, and LDL receptor-related protein 1 (LRP1)) [34,35]. This is important because synaptic turnover or pruning is critical for synaptic health. Conversely, APOE2 enhances the rate of phagocytosis of synapses by astrocytes and maintains a clean synaptic environment free from senescent synapses [35]. Additionally, glial activation is increased in mice expressing the APOE4 compared with APOE2 and APOE3. After lipopolysaccharide (LPS) injection, glial numbers and inflammatory markers are increased, while synaptic markers are decreased in APOE4-expressing mice [33]. However, it is important to note that APOE is necessary for normal glial activation and acts as an anti-inflammatory, as APOE knockout animals show a similar influx in inflammation as APOE4 mice [33]. In summary, APOE4 decreases phagocytosis and increases glial activation, potentially leading to an influx of inflammation and senescent cells in the AD brain [23,35]. This enhancement of glial activation most likely acts through LRP1 similar to the pruning mechanism. Taken together, these studies suggest that APOE is essential in regulating neuronal signaling, but that the APOE4 isoform negatively impacts these signaling systems.

## APOE’s role in AD

Besides contributing to Aβ and tangle formation, APOE has also shown to control cerebral metabolism and blood flow resulting in neuroinflammation. Because of the multifaced effects of APOE, interest has grown within the research community to identify the precise molecular mechanisms through which APOE contributes to the neuropathogenesis of AD. Hopefully, various therapeutic strategies for ameliorating pathogenic changes related to APOE will become evident as more pathophysiological relationships in AD are established. In this section, we will briefly discuss the role of APOE in amyloidogenesis and tauopathy irrespective of neuronal signaling, along with recently developed insight into the effects of APOE on autophagy and mitochondrial function.

### APOE and β-amyloid hypothesis

**Figure 1:**
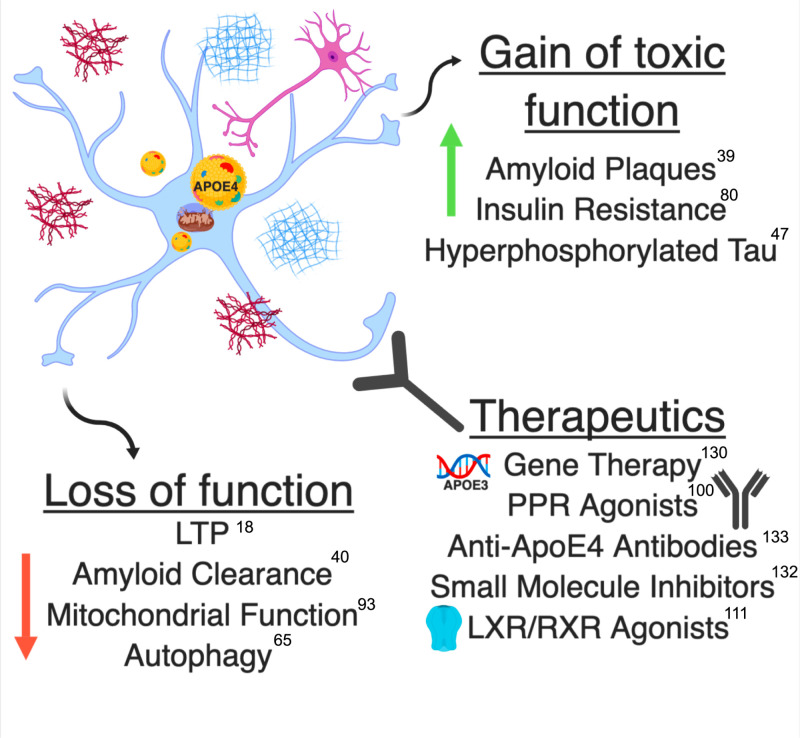
ApoE4’s impact on AD pathology ApoE4 has several gain of toxic function and loss of function roles. There is an increase in plaques, tangles, and insulin resistance. There is a decrease in LTP, amyloid clearance, mitochondrial function, and autophagy. Taken together, these changes result in neurodegeneration. Therapeutics such as, gene therapy, PPR agonists, anti-APOE4 antibodies, small molecule inhibitors, and LXR/RXR agonists could potentially slow or prevent neurodegeneration.

APOE plays a vital role in the metabolism of amyloid β (Aβ). APOE2, APOE3, and APOE4 proteins can each directly bind with Aβ, forming APOE/Aβ complexes that alter Aβ clearance, aggregation, and the formation of senile plaques [36]. APOE/Aβ binding efficiency is isoform specific, but reports of various affinities for Aβ are somewhat conflicting [37,38]. Notably, APOE4 reportedly binds with Aβ more aggressively than the other isoforms. In an AD mouse model overexpressing human APOE4 isoforms (PDAPP/TRE), Aβ load is increased in the brain interstitial fluid almost two- and four-fold higher than in APOE2- and APOE3-expressing mice, respectively. The increased deposition of Aβ also positively correlates with its rate of clearance [7]. Recent evidence indicates that increased expression of astrocytic apoE4, during the seeding stage of amyloid development, leads to increased amyloid deposition [39]. Moreover, APOE4 can also affect Aβ clearance through formation of APOE/Aβ complexes, as well as through competition for the same clearance pathway in the brain. When the same pathway is being utilized by both proteins, the overall rate of Aβ clearance is reduced [40]. Both APOE4 and Aβ compete for LRP1-dependent cellular uptake pathway in astrocytes. LRP1 facilitates Aβ uptake and degradation within cells by modifying cytoskeletal enzymes (e.g. matrix metalloproteinase-2 (MMP2) and MMP9) [39] and through phosphoinositide 3-kinase (PI3K)-extracellular signal-regulated kinase (ERK) pathways [41]. APOE4 competitively inhibits LRP1/Aβ binding, thus contributing to an overall increase in Aβ load (Figure 1). Because APOE regulates lipid metabolism and impaired lipid regulation correlates to increased Aβ production in the brain, there remains a general consideration of whether APOE additionally affects Aβ synthesis [42]. Aβ can conversely modulate APOE by affecting its internalization [43]. Specifically, in the presence of Aβ, both APOE3 and APOE4 bind with LDL receptors, undergo conformational changes, and exert significant internalization. This leads to increased binding to hippocampal neurons, more so for APOE4 than APOE3, and an overall increase in neuronal uptake of Aβ mediated by APOE4. The internalization and increased domain interaction of APOE4 with LDL receptors can also affect the intracellular amyloid precursor protein (APP) recycling process, contributing to a further increase in Aβ load via enhanced Aβ production [43].

Though the AD research field has shifted in recent years from the amyloid cascade hypothesis to focusing on τ, inflammation, metabolism, and hyperactivity, the impact of APOE4 on Aβ pathology still deserves attention as a potential target for disease-modifying treatment. Considering Aβ deposition can be noticed in the brain even decades before the onset of actual clinical symptoms [44], identification of this window period between the deposition and disease occurrence can serve as an opportunity for therapeutic intervention [45]. Instead of targeting amyloid using antibodies or trying to destroy plaque burden itself, future therapies could possibly inhibit APOE/Aβ competition or enhance Aβ clearance by increasing expression or altering functionality of LRP1 [46].

### APOE and τ pathology

**Figure 2:**
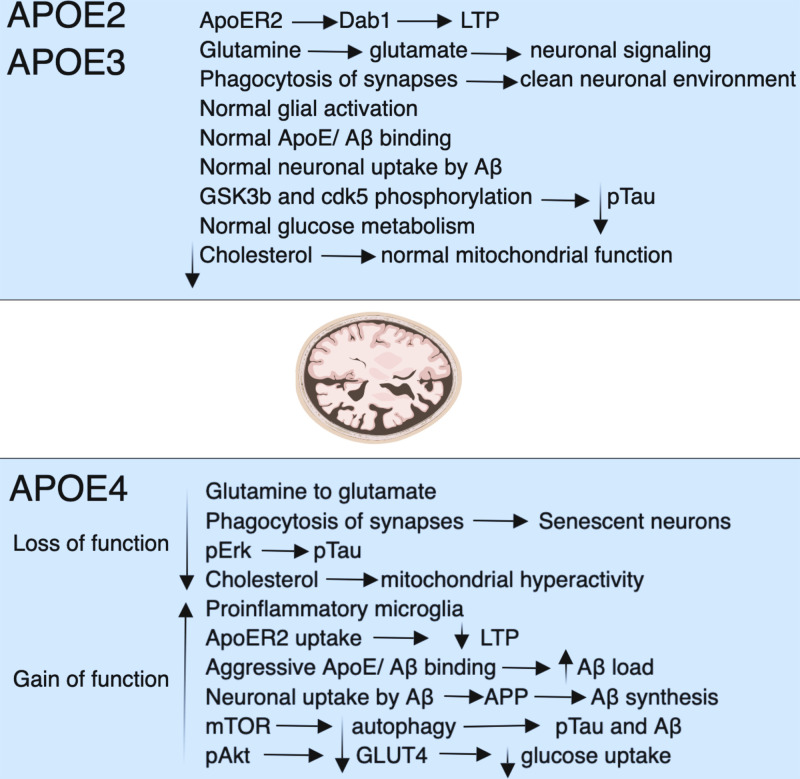
Summary of the mechanistic pathways affected by APOE4 APOE consists of three isoforms; APOE2, 3, and 4. APOE4 pathways tend to lead to neurodegeneration while 2 and 3 are protective. Specifically, there is a loss and gain of function that occurs with APOE4 carriers. Glutamine is not easily converted into glutamate, a loss of phagocytosis leading to older neurons, an increase in phosphorylated τ, proinflammatory microglia, mitochondrial hyperactivity, aggressive amyloid binding leading to plaques, and a decrease in long-term potentiation, autophagy and glucose uptake. Abbreviations: GLUT4, glucose transporter; mTOR, mammalian target of rapamycin; pAkt, phosphorylated pAkt; pERK, phosphorylated ERK; pTau, phosphorylated τ.

APOE is involved in microtubule formation and polymerization and thus promotes cytoskeletal integrity [47,48]. In normal physiological processes, neural outgrowth is stimulated by APOE3 and inhibited by APOE4 [49]. Presence of APOE4 in murine cell culture affects tubulin polymerization and microtubular morphology [50], resulting in microtubular depolymerization and problems with neuronal remodeling later in life. Hyperphosphorylation of microtubule-associated protein τ leads to the formation of neurofibrillary tangles [51], one of the two hallmarks of AD. Emerging research suggests that APOE4 interaction with τ can be more damaging than the APOE4–Aβ interaction [52]. As recent as 2010, researchers reported that increased Aβ in cerebrospinal fluid (CSF), not τ protein, was associated with APOE4 genotypic individuals [53]. Recent post-mortem analysis of AD brains revealed a more significant association of APOE4 with τ filaments and tangles exists in the presence of brain Aβ than in the absence of brain Aβ [54], suggesting that APOE4-mediated effects on τ may be Aβ-dependent. However, the APOE-mediated influence on τ pathology can be independent of Aβ as well. Genetically modified P301S transgenic mice with APOE4 knockin exhibit increased τ levels in brain compared with *APOE* knockout mice, also accompanied by neuroinflammation and cerebral atrophy [52] thought to be mediated by increased microglial activation, leading to greater secretion of proinflammatory cytokines. APOE4 has been also linked to other tauopathies such as corticobasal degeneration (CBD), progressive supranuclear palsy (PSP), and Pick’s disease strengthening the fact that APOE can regulate the τ in both Aβ-dependent and -independent ways. Notably, post-mortem analysis revealed that, in individuals suffering from sporadic primary tauopathy, ε4 allele carriers manifested with more severe regional neurodegeneration [52]. Increases in hyperphosphorylated τ in APOE4 overexpressing transgenic mice model has also been shown to be age-dependent and to correlate with behavioral deficits [47]. Interestingly, an increase in p-τ within the hippocampus and cortex has been associated with increased phosphorylation and activation of ERK [55], indicating possible APOE4, p-τ, and p-ERK interplay (Figure 2). Though this study observed no relationship between APOE4 and other downstream kinases such as glycogen synthetase kinase (GSK3β), cyclin-dependent kinase-5 (CDK5), and its activators, others have reported that APOE4 can additionally mediate τ phosphorylation via these kinases [56,57]. In concordance, application of APOE2 in cell culture increases protein kinase B (Akt) and ERK activation but reduces activation of c-Jun N-terminal kinases (JNK). The APOE2 treatment of cells is also associated with reduced GSK3β and CDK5 phosphorylation [58], corresponding to a decreased level of phosphorylated τ. Conversely, in the presence of lower APOE2 levels, increased GSK3β phosphorylation has been associated with increased site-specific τ phosphorylation at Thr^172^, Ser^387^, and Ser^395^ [59]. Studies suggest that calcium may play an additional role in this Akt-ERK-GSK3β signal transduction cascade, as treatment of primary cell culture with APOE peptide showed extracellular calcium influx and subsequently elevated cytosolic calcium levels [60]. This can in turn activate several downstream kinase signaling pathways, leading to site-specific τ dephosphorylation at Thr^231^, Ser^235^, and Ser^396^ [61].

Although the relationship between APOE and τ phosphorylation is now somewhat established, further investigation of isoform-specific effects of APOE on τ protein remains crucial. Since APOE can both positively and negatively influence τ regulation and modulate AD pathology accordingly, future studies should be heavily focused on how each isoform of APOE regulates τ function rendering microtubular and cytoskeletal instability contributing to the disease course.

### Autophagy and AD

Autophagy is a physiological process through which cells dispose of damaged organelles to make room for newly regenerated ones. The role of autophagy in AD is currently being heavily investigated since proper autophagy and lysosomal functioning is necessary for successful degradation of Aβ and τ to hinder the formation of plaques and tangles. Autophagy is impaired from the early stages of AD [62–64], and recent evidence suggests that APOE can influence autophagic flux and thereby affect the pathological course of AD. However, the mechanisms leading to autophagy dysfunction in AD need to be elucidated. The controversial question is whether autophagy or lysosomal function plays more of a direct role. While Boland et al. [65] suggest that autophagy is not directly involved in the regulation of APP metabolism, Barbero-Camps et al. [66] demonstrates that higher brain cholesterol enhances autophagosome formation and disrupts its fusion with endosomal–lysosomal vesicles. These studies suggest that both autophagy and the lysosome are playing roles in APP degradation. Furthermore, reduced levels of autophagy-related mRNA is observed in the post-mortem analysis of hippocampal tissue from AD patients with APOE ɛ4/ɛ4 genotypes [4,67]. Competitive binding of APOE4 to ‘coordinated lysosomal expression and regulation’ (CLEAR) DNA motifs can result in suppression of autophagy leading to accentuation of AD-like pathology [67]. Additionally, a study of cortex and hippocampal samples from AD patients with similar genotypes revealed increased intraneuronal Aβ accumulation [68]. In accordance with this, genetic ablation of APOE4 largely improved the previously observed damages including neuronal loss and cerebral atrophy. The major protein involved in cellular autophagic processes is the mammalian target of rapamycin (mTOR), which is generally responsible for maintaining homeostasis of cell growth and cell death. mTOR overactivation compromises autophagy, leading to τ hyperphosphorylation and reduced Aβ clearance [69] (Figure 2). Inhibition of mTOR by rapamycin increases the expression of autophagic markers in brains of transgenic P301S τ mice, resulting in improved behavioral outcomes [70]. Rapamycin treatment in APOE transgenic mice also attenuates neuroinflammation by improving cerebral blood flow through activation of endothelial nitric oxide synthetase [71], thereby helping to maintain integrity of the BBB otherwise compromised in AD [72]. APOE4-expressing astrocytes show reduced degradation of Aβ in the 5xFAD mouse model, which is improved in the presence of the autophagy inducer rapamycin [73]. Although rapamycin was thought to promote insulin resistance, recent evidence suggests that rapamycin produces benefits in energy metabolism in the context of Type II diabetes. However, this mainly depends on the pancreatic insulin content (PIC) of the animal or individual. For example, models with low PIC exhibited greater beneficial effects than models with high PIC [74,75]. It is notable that rapamycin has been shown to effectively block the progression of AD by restoring the neurovascular and neurometabolic functions, which may make rapamycin a good choice as a preventive therapy for pre-symptomatic AD [70]. Last, rapamycin extends the lifespan of AD rodents’ models [76,77] and further substantiates consideration of this pathway in AD treatment.

In summary, rapamycin, a common immunosuppressive drug used in organ transplantation, is currently undergoing pre-clinical investigation as an alternative pharmacotherapeutic for AD. Because APOE4 is the greatest genetic risk factor for AD and rapamycin can modulate the adverse physiologic consequences of APOE4, this agent could be considered as a prophylactic measure for APOE4 carriers. Future studies should be conducted to evaluate the ability of rapamycin to prevent or slow AD disease progression long term, with treatment beginning in very early stages perhaps in a humanized APOE model also exhibiting amyloid and τ pathology.

### Role of APOE in brain insulin signaling and glucose metabolism

AD has unofficially been referred to as Type III diabetes due to its relationship with reduced insulin secretion and higher insulin resistance in the brain [78]. This classification is strengthened by results from a small clinical trial in which intranasal insulin improved behavioral outcomes of early AD patients and inhibited progression of cognitive decline [79]. Interestingly, APOE is thought to be critically involved in glucose utilization in brain. In a prospective cohort study, elevated blood glucose levels were observed in middle-aged APOE4 carriers who exhibited neuropathological changes consistent with AD upon post-mortem examination [80] (Figure 1). Introduction of the human *APOE4* gene in APP transgenic mice impairs phosphorylation of Akt downstream from insulin receptor signaling, which may be attributable to insulin resistance (Figure 2). Dysfunction of insulin signaling in this model is also associated with impaired spatial memory [81], as well as impaired glucose uptake, as indicated by reduced levels of glucose transporter GLUT4 [82]. In comparison with APOE2-expressing mice, those expressing APOE4 exhibit significant reductions in GLUT4, insulin-like growth factor 1 (IGF-1), insulin receptor substrates, and insulin-degrading enzyme (IDE), which indicates differential regulation of brain glucose metabolism by APOE2 and APOE4 isoforms. Neuronal APOE4 interferes with insulin receptor trafficking by trapping the receptor to endosomes, ultimately leading to altered glycolysis [83]. APOE also impairs trafficking of other membrane-bound receptors such as LRP1. This neuronal membrane receptor, mentioned earlier for its role in Aβ clearance, has also been implicated in glucose metabolism. Conditional LRP1 knockout mice exhibit insulin resistance via the aforementioned impaired glucose regulating mechanisms [84]. On the other hand, exogenous insulin supplementation increases neuronal LRP1 expression, thereby correcting the brain glucose intolerance. Insulin administration also improves synaptic plasticity and behavioral outcomes in diabetic mice [85], and investigating whether insulin attenuates Aβ-mediated impairments in synaptic plasticity would be a promising topic for future studies.

Although evidence suggests that peroxisome proliferator-activated receptor γ (PPARγ) modulators like thiazolidinediones (TZD) improve neuroinflammation and synaptic plasticity [86], the detailed mechanism for these effects is yet to be explored [87]. However, insulin is generally regarded as safer due to the adverse effects of TZDs on other organ systems. Another interesting approach to possibly increase glucose uptake in the brain is through modulation of cannabinoid receptor type 2 (CB2R). Since CB2R exhibits anti-inflammatory effects, its role in neuroinflammation in the context of AD should be explored further. Oral cannabinoid administration reduces cortical Aβ and improves behavioral performance in a mouse model of AD [88]. CB2 agonists also enhance glucose uptake in brain and promote proper glucose utilization [89], but using cannabinoids as an AD therapy is possibly counterintuitive since activation of cannabinoid receptor type 1 (CB1R) by non-selective cannabinoid receptor agonists can trigger learning and memory deficits [90].

In summary, the involvement of glucose metabolism dysregulation in AD is relatively well established, and improving insulin sensitivity and glucose utilization would likely improve hallmarks of the disease. Since APOE4-mediated insulin resistance can contribute to impaired cognition and dementia, targeting the glucose metabolic pathways to reduce the glycemic load in brain could be an alternative therapeutic approach to mitigate the APOE4-mediated effects. Specifically, while APOE4 carriers exhibit lower glucose tolerance even before developing the pathological signs of AD, epigenomic modifications of the *APOE4* gene, and its effect on glucose metabolism can also serve as a basis for identifying future preventive strategies. However, it is necessary to evaluate critically the above-mentioned therapeutic strategies in order to find proper therapeutic windows to avoid adverse effects.

### APOE and mitochondrial function

Alterations in mitochondrial turnover and hypometabolism have been observed in AD [91]. For example cytochrome *c* oxidase, one of the major mitochondrial energy regulators, is significantly reduced in the hippocampus and temporal cortex of AD patients [92]. A previous study in humans revealed significant associations between serum APOE level and lipid peroxidase levels in the E 4/3 phenotype but not the E 3/3 or E 3/2 phenotypes [93]. Since APOE affects mitochondrial protein activity in neuronal cells, recent studies have investigated whether disruption of mitochondrial homeostasis by APOE is involved in the pathogenesis of AD. APOE3-containing astrocytes prepared from human APOE3 knockin mice release more cholesterol than APOE4-containing astrocytes from human APOE4 knockin mice. Thus, APOE3 can exert a protective mechanism for AD via decreased cellular cholesterol, Aβ production, and formation of neurofibrillary tangles [94]. Conversely, hyperactivity of mitochondria‐associated endoplasmic reticulum proteins is present in APOE4-treated astrocytes [95] (Figures 1 and 2). These proteins are specifically designated for cholesterol transport, and alteration of their function leads to an imbalance in lipid-mediated cellular processes. The aforementioned effect is also more significant in APOE4-treated cells than those treated with APOE3. Although APOE4 is generally associated with a higher risk for AD, certain APOE4 fragments (1–272) might exert a more profound effect on mitochondrial respiratory complex dysfunction than intact APOE4 molecules (1–299) [96]. Increases in reactive oxygen species (ROS), lactose metabolism, and subsequently oxidative stress is also observed in APOE4-treated PC12 cells [97]. These effects may be due to specific APOE4 domain interactions, mediated by Arg^61^ in the N-terminal domain and/or Glu^255^ in the C-terminal domain [98]. This pathological domain interaction is responsible for a decrease in the phospholipid-binding capacity of APOE4, resulting in reduced cholesterol secretion from APOE4-producing neurons. Interestingly, mutation from Arg^61^ to Thr restores observed mitochondrial respiratory complex dysfunction, while mutation from Glu^255^ to Ala disrupts the domain interaction [98], suggesting possible future therapeutic strategies [99].

Another important consideration in AD pathology is abnormalities in mitochondrial biogenesis, the process through which balance between mitochondrial energy production and expenditure is maintained. Peroxisome proliferator-activated receptor γ coactivator-1α (PGC-1α) is also a major player in mitochondrial biogenesis and regulates various mitochondrial functions at both the transcriptional and post-transcriptional levels [91]. Post-mortem analyses of brains from AD patients show a reduced amount of PGC1-α, and exogenous PGC1-α application reduces Aβ levels in neuronal culture [100]. However, the role of PGC1-α in relation to mitochondrial function in AD has not been fully elucidated and demands further attention to be established as an alternative therapeutic target. Since PPAR agonists like TZDs up-regulate PGC1-α in various tissues like the heart [101] and skeletal muscle [102], these drugs should be further investigated for their effect on PGC1-α in brain as a possible mechanism to alleviate APOE-mediated mitochondrial dysfunction.

In summary, the role of APOE as a major modulator of cellular lipoprotein and cholesterol transport process should not be overlooked, as these are invariably altered in AD. Although APOE alters mitochondrial function, in-depth mechanisms are still not clear. However, addressing homeostatic imbalance of mitochondrial function via the alternative therapeutic strategies discussed above presents a promising future for AD treatment. Nonetheless, future research should be focused on translating *in vitro* observations related to intricate molecular mechanisms to *in vivo* animal studies for observations of how these mechanisms affect behavioral outcomes and disease progression.

## Current therapies modifying APOE-mediated AD pathology

While APOE status often serves as a risk assessment and recruitment tool for AD drug therapy trials, very few attempts to actively target this lipoprotein have been made. In this section of the review, we hope to shed light on the benefits and challenges of targeting APOE in the prevention and treatment of AD. Briefly, we reviewed a number of possible pathways through which APOE modifies disease progression (Figure 2), and we will now discuss current therapies in clinical trials that directly target these pathways, current therapies directly targeting APOE, and possible future directions for APOE-related therapies for AD (Figure 1).

As previously discussed, APOE status seemingly alters multiple physiologic processes like Aβ deposition, hyperphosphorylation of τ and development of neurofibrillary tangles, autophagy, insulin signaling and glucose metabolism, and mitochondrial function. Currently, numerous therapies targeting these alterations are undergoing clinical trials and could potentially alleviate disease progression by addressing downstream dysregulations influenced by APOE physiology. More specifically, 14 AD drug therapies in current Phase III clinical trials target amyloid pathology, and 1 targets tauopathy. As far as other agents in Phase III trials working through pathways discussed in this review, only brain insulin signaling is being targeted through the use of intranasal insulin administration. However, numerous other therapies under pre-clinical, Phase I, and Phase II investigations target the other pathways discussed above. A comprehensive review of AD therapies undergoing clinical trials in 2018 was previously published by Cummings et al. [103], and thus, for the remainder of this review, we will briefly focus on a few clinical and pre-clinical investigative therapies that specifically affect APOE physiology or directly target the lipoprotein molecule.

### AD risk and lipid-modifying medications

Of the anti-AD therapies reviewed by Cummings et al. [103], only one hopes to influence APOE physiology directly through the use of a dated and discontinued lipid-modifying drug called probucol [104]. While the exact mechanism of probucol is unknown, it exhibits antioxidant properties and may inhibit the early stages of cholesterol synthesis, as well as interfere with intestinal absorption of cholesterol [105]. Additionally, probucol alters LDL catabolism and is still being studied for its benefits in cardiovascular disease [106–112]. In a small pilot study over a decade old, researchers found that probucol increases both serum and CSF APOE concentrations, which correlated to decreased p-τ and Aβ burden in AD patients [113]. This observation lead to larger clinical trials, the most recent of which has been completed but not yet published. Probucol repurposing in the treatment of AD is interesting but unsurprising when considering the proposed impact of lipoproteins on disease progression, and it is not the only lipid-lowering therapy to be assessed in AD patients. Though having promising clinical outcomes in its prime, probucol was completely replaced by modern-day statin therapy [114], the gold-standard for treating high cholesterol and reducing cardiovascular risk.

Statins, or 3-hydroxy-3-methyl-glutaryl-coenzyme A (HMG-CoA) reductase inhibitors, have also been repeatedly evaluated for their possible role in AD prevention and treatment [115–117]. While these medications produce variable results, they are generally regarded as beneficial in terms of AD risk reduction [118,119]. Though observed in multiple populations [120,121], statin effects on cognitive function are not currently factored into prescribing considerations. Additionally, statin therapy seems to be limited to AD risk reduction with no consistently significant effects on delay of AD progression or on pathological hallmarks in diagnosed AD patients regardless of APOE status [122]. These observations indicate that statin benefit in AD may be largely related to decreasing the risk for brain vascular changes that are commonly found along with AD brain changes [1]. Vascular dementia is the second most prevalent type of dementia, is a significant risk factor for development of AD, and is more likely in patients harboring both *ε4* alleles [123–125].

Other commonly prescribed lipid modifiers, like omega-3 fatty acids, have been supplemented for AD prevention and treatment with variable results [126,127]. Because omega-3 is neuroprotective *in vitro* [128,129], its potential use in AD was accordingly investigated. Changes in cognition as measured by mini-mental state examination (MMSE) and AD assessment subscale (ADAS-cog) are similar in active treatment and placebo groups, but a trial duration of only 6 months is unlikely to produce measurable differences in cognition. Interestingly, supplementation with omega-3 for 6 months is associated with a decrease in epigenetic markers in immune cells, and the authors postulate that these modifications could possibly reduce inflammatory mechanisms in AD [130]. However, these changes occur independent of APOE4 status [130]. Most studies analyze effects of lipid therapies relative to APOE status, where APOE4 carriers typically exhibit high LDL, the ‘bad cholesterol’, and low high-density lipoprotein (HDL), the ‘good cholesterol’. However, the complex interaction of multiple diseases within the elderly and AD blurs the path to definitive monotherapy one might otherwise hope to pursue. Even considering the increased prevalence of diabetes, dyslipidemia, or stroke in those also at a high risk for developing AD [131], the APOE4 genotype still presents an interesting correlate to the impact of various comorbidities on relative risk for dementia.

Much like challenges faced with other pharmacotherapies, difficulty in targeting the APOE lipoprotein stems from its role in normal physiology as previously discussed [12, 13, 14]. Specificity for both pathologic APOE and site of action in the body will be crucial in future development of AD therapies that avoid potentially harmful dysregulation of lipid transport. Thus, developing AD therapies that directly target specific APOE lipoproteins rather than indirectly affecting the downstream effects of APOE will more definitively quantitate the magnitude of APOE-mediated detriments attributable to allelic variations in the presence of comorbidities. Along with further quantitation of APOE’s relative impact on AD risk, directly targeting these lipoproteins could predictably treat an underlying ‘root-cause’ of AD progression.

### Therapies directly targeting APOE lipoproteins

Currently, the most interesting AD clinical trial aiming to directly target APOE is a Phase I assessment of the safety and toxicity of gene therapy in APOE4 homozygotes [132]. In hopes of eventually altering the compounded risk of the APOE4/APOE4 genotype in AD, this trial will assess the maximum tolerable dose of APOE2 cDNA adeno-associated virus (AAV) gene transfer via intracisternal administration over 2 years. Researchers hope to shift APOE lipoprotein isoform expression from APOE4 to APOE2/APOE4 using this method, thus conferring neuroprotective effects of the APOE2 lipoprotein while simultaneously decreasing the impact of APOE4-mediated detriments in AD. Unfortunately, this trial has not begun the recruitment phase, and we cannot comment on observations other than those from the promising pre-clinical animal studies that motivated transition into human clinical trials.

Much like the results from pre-clinical animal studies assessing APOE-AAV gene transfer [133], two recent pre-clinical assessments of APOE-directed therapies have produced promising results. First, researchers developed a novel peptide antagonist designed to inhibit the Aβ/APOE4 interaction [134]. In doing so, APP/PS1 mice treated with the peptide show significant beneficial changes to soluble and insoluble Aβ peptide and oligomers. These changes are accompanied by significant cognitive improvements, as well as amelioration of Aβ pathology in the cortex and hippocampus. A second method using anti-APOE3 and APOE4-specific antibodies (HAE-4) similarly reduces Aβ pathology in APP transgenic mice [135]. This treatment does not significantly alter levels of APOE in plasma or the brain, but both direct central administration of the antibody and AAV-induced expression significantly reduce plaque accumulation *in vivo*. Unfortunately, the present study did not assess changes to behavior or cognitive function. While applicability is limited when compared with other *in vivo* studies, similar alterations to pathological hallmarks could presumably result in similar benefits to cognitive function.

In general, targeting APOE directly yields promising results in amelioration of pathological markers of AD and behavioral deficits. However, challenges of these therapies may arise not in efficacy of their mode of action, but rather the difficulty of replicating optimal delivery and avoiding potentially life-threatening adverse reactions. Delivery of large peptides or viral particles directly to the CNS remains a significant limitation to treat neurologic diseases. First, administration limitations may be overcome by drug delivery via intranasal route, which bypasses the BBB and produces significant brain distribution of large molecules and peptides [136–139]. This method has been used in past clinical trials and is currently being exploited in the Phase III SNIFF trail assessing the efficacy of intranasal insulin in patients with AD [140]. Alternatively, intracerebroventricular (ICV) injection is an undesirable route of administration for humans, but optimized inducible gene expression will significantly reduce the need for chronic frequent dosing. However, viral gene delivery carries with it the risk for life-threatening systemic inflammatory response, other immunologic reactions (i.e., CD8-mediated attack of affected cells), and possible genotoxicty in the rare event of AAV cDNA/host DNA integration [141,142]. However, AAV gene delivery is a consistently growing field with current simultaneous aims of optimizing safety and establishing replicable efficacy from animal models to humans. At last, recent development of APOE small molecule effectors may eventually produce therapeutics with more ideal BBB penetration following systemic administration [143,144], but these agents have only been assessed *in vitro* and require more extensive evaluation in animal models.

## Conclusion

Mounting evidence suggests that APOE4 contributes to AD pathogenesis through multiple pathways. The work summarized in this review highlights the relation between APOE and deficits in cognitive functioning within the context of APOE’s role in neuronal functioning and signaling, AD-related pathology including β-amyloid and τ, autophagy, brain insulin signaling and glucose metabolism, and mitochondrial functioning. The limitations of therapies targeting these pathways or APOE directly, and possible future directions for APOE-related therapies for AD, were also discussed. Together, the summarized work suggests APOE is a promising target for therapy and drug development against AD, but the co-occurring ‘gain-of-toxic functional’ and ‘loss-of-functional’ profiles of APOE4 in different systems may make developing effective APOE-targeted therapies for AD difficult (Figure 1). A greater understanding of the synergistic roles of APOE in multiple pathways is critical, as well as an understanding of the therapeutic windows for each pathway.

## Abbreviations

AAV: adeno-associated virus
AD: Alzheimer’s disease
Akt: protein kinase B
APOE: apolipoprotein E
APOER2: APOE receptor 2
APP: amyloid precursor protein
BBB: blood–brain barrier
CB2R: cannabinoid receptor type 2
CDK5: cyclin-dependent kinase-5
CNS: central nervous system
CSF: cerebrospinal fluid
EC: entorhinal cortex
ERK: extracellular signal-regulated kinase
GSK3β: glycogen synthetase kinase
LDL: low-density lipoprotein
LRP1: LDL receptor-related protein 1
mTOR: mammalian target of rapamycin
NMDA: N-methyl-D-aspartic acid
PDAPP/TRE: platelet derived growth factor + amyloid precursor protein
PIC: pancreatic insulin content
PNS: peripheral nervous system
PS1: Presenilin 1
TZD: thiazolidinedione
